# Cervical disc prostheses need a variable center of rotation for flexion / extension below disc level, plus a separate COR for lateral bending above disc level to more closely replicate in-vivo motion: MRI-based biomechanical in-vivo study

**DOI:** 10.1186/s12891-022-05121-2

**Published:** 2022-03-08

**Authors:** Manfred K. Muhlbauer, Ernst Tomasch, Wolfgang Sinz, Siegfried Trattnig, Hermann Steffan

**Affiliations:** 1 Neurosurgical Department, Klinik Donaustadt, Langobardenstrasse 122, 1220 Vienna, Austria; 2 Vehicle Safety Institute, Graz University of Technology, Graz, Austria; 3 High Field MR Center, Department of Biomedical Imaging and Image-guided Therapy, Medical University of Vienna, Vienna, Austria

**Keywords:** Cervical disc prostheses, Cervical arthroplasty, Cervical spine biomechanics, In-vivo kinematic study

## Abstract

**Background:**

Cervical disc prostheses are used to preserve motion after discectomy, but they should also provide a near-physiological qualitative motion pattern. Nevertheless, they come in many completely different biomechanical concepts. This caused us to perform an in-vivo MR-based biomechanical study to further investigate cervical spine motion with the aim to gain new information for improving the design of future cervical arthroplasty devices.

**Methods:**

Fifteen healthy volunteers underwent MRI-investigation (in order to avoid radiation exposure) of their cervical spines from C3 to C7; for each segment centers of rotation (COR) for flexion / extension were determined from 5 different positions, and CORs for lateral bending from 3 different positions. The motion path of the COR is then described and illustrated in relation to the respective COR for maximum flexion / extension or lateral bending, respectively, and the findings are translated into implications for a better biomechanical prosthesis-design.

**Results:**

The COR for flexion / extension does not remain constant during motion. The CORs for the respective motion intervals were always found at different positions than the COR for maximum flexion /extension showing that the COR moves both along the x- and the y-axis throughout flexion / extension. For lateral bending a completely independent COR was found above disc-level.

**Conclusion:**

Flexion / extension is not a simple circular motion. Disc prostheses need a variable COR for flexion / extension below disc level with the capability to move both along the x- and the y-axis during motion, plus a second completely independent COR for lateral bending above disc level to closely replicate in-vivo motion. These findings are important for improving the biomechanical design of such devices in the future.

## Introduction

Biomechanical studies investigating cervical spine motion and describing how the cervical vertebral bodies exactly move against each other in healthy subjects are essential for the design of cervical disc prostheses.

A large variety of such prostheses are – or have previously been - available on the market for preserving motion after discectomy. However, they significantly differ in design and position of the COR. Some have one fixed COR below the motion segment (ProDisc C, DePuy-Synthes; PCM, Cervitech; Discover, DePuy-Synthes), others a COR above the motion segment level with capability for additional sagittal translation (Prestige STLP, Medtronic), again others a variable COR (Bryan, Medtronic) or no articulating surfaces at all but an elastic nucleus (M6, Spinal Kinetics). An analysis of the motion pattern after insertion of a Bryan-, a Prestige STLP or a Discover prosthesis in patients after cervical discectomy could show that a more flexible biomechanical design can contribute to a better physiological motion [1].

The great variety of biomechanical studies available include in vitro and in vivo studies using different techniques like plain flexion-extension radiographs [2–5], biplanar radiography [6], cinematography [7–9], CT [10–13] or three-dimensional-MRI [14, 15]. Many of these studies focus upon the range of motion (ROM), some upon the description of coupled motion in rotation, lateral-bending and flexion / extension [6, 14, 15]. Less information is available regarding how the respective vertebral bodies exactly move against each other. It is known that the center of rotation (COR) in flexion / extension varies from segment to segment, and that the COR for lateral-bending does not correspond to the COR for flexion / extension [9, 16–19]. Especially the studies from Anderst and Baillargeon investigate the trajectory of the COR during flexion / extension more precisely and describe level-specific differences both in location and motion-path of the COR [20–22]. Moreover, a precise three-dimensional view is given in these studies how the cervical vertebral bodies move against each other in coupled lateral bending / rotation. But also these excellent studies do not describe the motion-path of the COR in a manner that allows translation of their findings into clear recommendations how the design of cervical disc prostheses can be improved in order to more closely replicate in-vivo motion. Therefore, we conducted an in vivo MRI-based biomechanical study investigating the motion from C3 to C7 in healthy volunteers in order to describe the motion trajectory of the COR in flexion / extension and lateral bending.

### Materials and methods

Fifteen healthy volunteers (6 male, 9 female; age 25y – 53y; mean-age 37.5y) with no previous symptoms of cervical spondylosis underwent MRI-investigation of their cervical spines after giving informed consent to the study protocol which was approved by the Ethic commission of the Medical University of Vienna (EK Nr. 571/2007).

All investigations were done using a 1.5 T MRI (Siemens Avanto 1.5 T; Siemens Erlangen, Germany).

### Flexion / extension

Flexion / extension was recorded in 5 different positions for each volunteer. Data for flexion / extension were collected from the following positions: Maximum extension (ME), intermediate extension (IE), neutral position (N), intermediate flexion (IF) and maximum flexion (MF). The volunteers were asked to actively move their heads into these positions and were then supported with cushions to remain in the respective position during MRI data acquisition. The cushions were used to have similar positions of the head for all volunteers only. The motion-amplitudes between these 5 different positions were approximately 3^o^. T2-weighted median-sagittal slices showing the entire contours of the vertebral bodies C3 to C7 were used for biomechanical calculations. This protocol allowed acquisition of 5 different data sets during maximum flexion and extension and therefore enables the determination of 4 different intermediate CORs for each motion step (ME-IE, IE-N, N-IF, IF-MF). In addition to these intermediate CORs, also for the entire motion between ME and MF a total COR can be calculated. The x / y-coordinates of the intermediate CORs are compared with the x / y-coordinates of the COR for total flexion / extension, and the motion path of the COR is illustrated in relation to the COR for total flexion / extension.

### Lateral-bending

Lateral-bending was recorded in 3 different positions, therefore receiving data from 2 separate motion amplitudes of approximately 4^o^. The volunteers were asked to actively move their heads to maximum left- and right-bending and were supported with cushions to remain in the respective position. T2-weighted frontal MRI-sections were collected showing the entire contours of the vertebral bodies C3 to C7 for biomechanical analysis. The respective z / y-coordinates of the CORs were analyzed for detection of significant changes during lateral-bending.

### Coordinate system

**Fig. 1:**
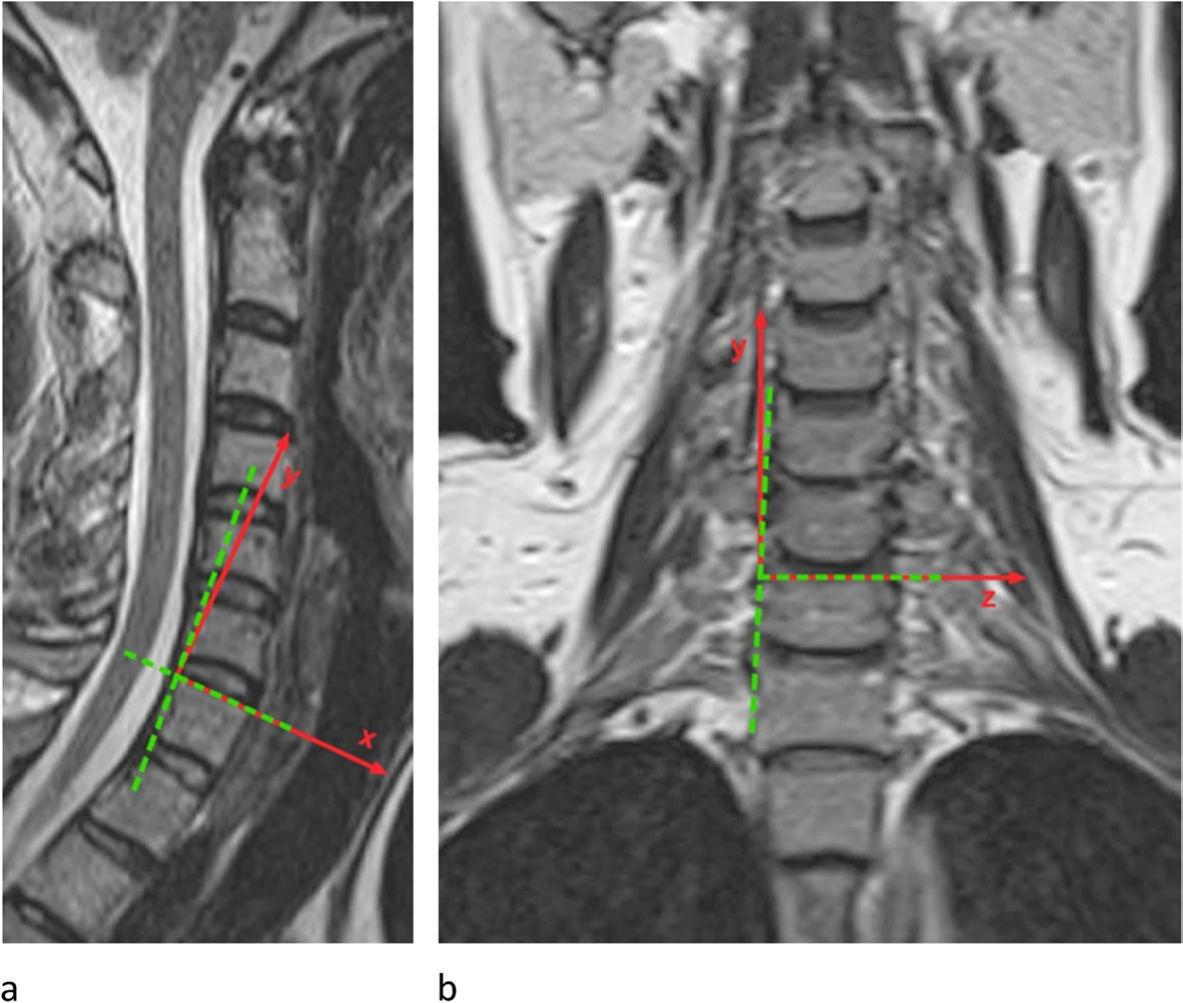
Definition of the coordinate-system **a** flexion / extension **b** lateral-bending

The coordinate-system for motion analysis of flexion / extension was determined using a line through the most superior anterior and the most superior posterior point of the respective vertebral body. The cutting point with a second line through the most posterior inferior and the most posterior superior point of the respective vertebral body was defined as the center of the coordinate-system with the x-axis passing through the most superior anterior point of the respective vertebral body, the y-axis directing cranially rectangular to the x-axis, and the z-axis rising orthogonally against the viewer (Fig. 1a). For lateral-bending the center of the coordinate-system was determined by the cutting point of the line through the two most superior points of the respective vertebral body with the line through the two most lateral points (Fig. 1b).

### Center of rotation and motion analysis

**Fig. 2:**
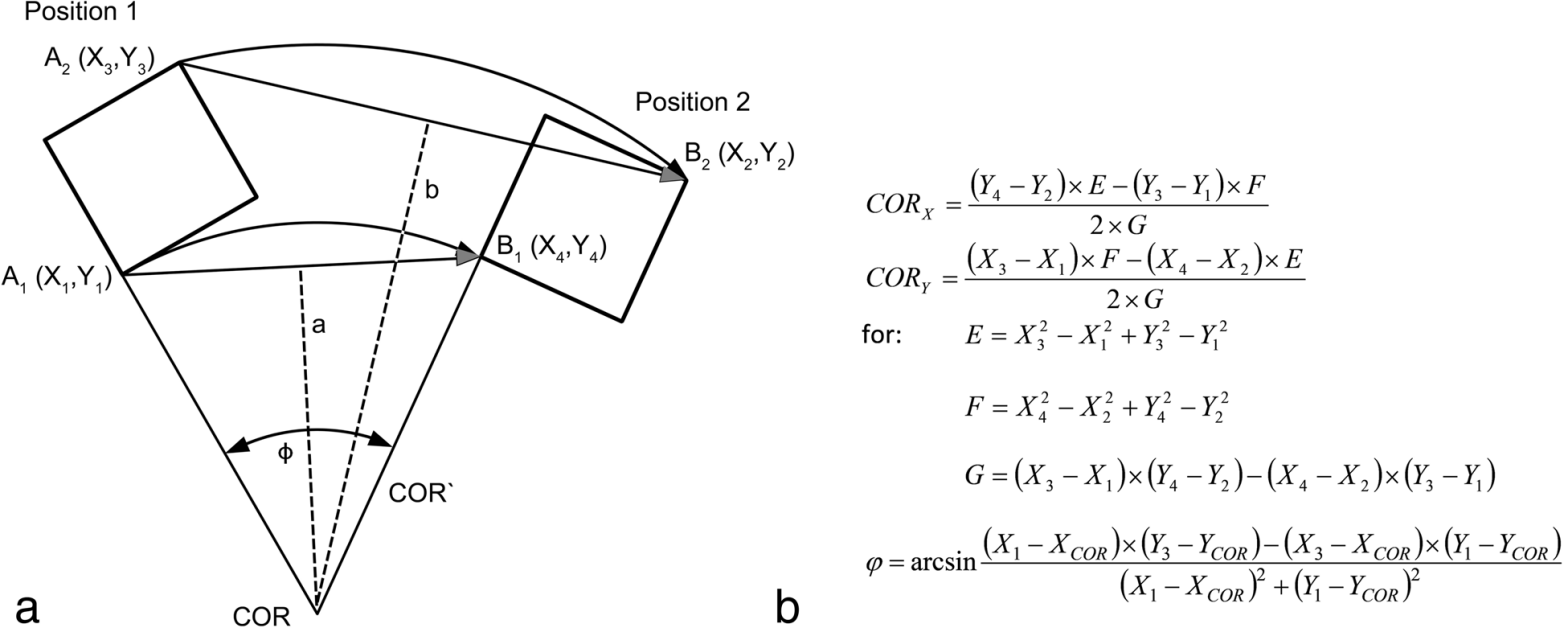
Determination of the COR. **a** graphically illustrates the determination of the COR; **b** shows the respective mathematical algorithm [23–25]

Figure 2a illustrates the determination of the COR. The COR for the 2 reference-points A and B is defined as the cutting-point of the 2 perpendicular bisectors a and b. This resulting COR is related to the 2 given end-positions of the vertebral body. Figure 2b shows the mathematical algorithm for determination of the COR [23–25].

To capture the coordinates of the reference points A and B the software AutoCAD (AutoCAD, AUTODESK, San Rafael, Ca, USA) was used. Data processing from MRI pictures to AutoCAD software was done by one single investigator; this required marking of the 4 corner-points of the respective vertebral bodies on the MRI pictures in order to create a quadrangle covering the vertebral bodies to allow better overlay of the respective vertebral bodies and to use all 4 edge-points of the quadrangle for a more precise calculation of the COR. Coordinate-calculation was done using Microsoft Excel software (Microsoft Excel, Microsoft, Redmont, Washington, USA). In flexion / extension both the COR from maximum extension to maximum flexion was calculated as well as 4 more CORs from the respective motion-steps in between. In lateral-bending, the COR from maximum left- and right-bending was calculated as well as 2 more CORs from the respective steps between left-to-neutral and right-to-neutral-bending.

### Statistical analysis

The t-test was used for determination of significance regarding the differences between the respective data-sets with a significance level of α = 0.05.

## Results

### ROM flexion / extension and lateral bending

Fifteen datasets were analyzed; the mean ROM for flexion / extension from C3 to C7 was 53.4^o^ (SD 12.7). Table 1 shows the mean values for maximum flexion / extension of the respective motion-segments compared to previously reported data in the literature. The mean-ROM found for lateral bending from C3 to C7 was 33.7^o^ (SD 9.9). Table 2 shows the mean-values between neutral position and maximum lateral bending of the respective motion-segments compared to previously reported data in the literature.

**Table 1:** ROM; Mean-values for maximum flexion / extension of the respective motion-segments compared to previously reported data in the literature

Study	ROM ^**o**^ (SD)				
	C2/C3	C3/C4	C4/C5	C5/C6	C6/C7
Aho et al. [26]	12 (5)	15 (7)	22 (4)	28 (4)	15 (4)
Bhalla and Simmons [27]	9 (1)	15 (2)	23 (1)	19 (1)	18 (3)
White and Panjabi [25]	8	13	12	17	16
Lind et al. [4]	10 (4)	14 (6)	16 (6)	15 (8)	11 (7)
Dvorak et al. [2]	10 (3)	15 (3)	19 (4)	20 (4)	19 (4)
Our study	–	11.4 (3.4)	14.9 (4.8)	12.7 (3.4)	14.4 (5.8)

**Table 2:** ROM; Mean-values between neutral position and maximum lateral bending of the respective motion-segments compared to previously reported data in the literature

Study	ROM ^**o**^ (SD)				
	C2/C3	C3/C4	C4/C5	C5/C6	C6/C7
White and Panjabi [25]	5	5,5	5,5	4	3,5
Panjabi et al. [6]	4.8 (0.9)	4.5 (1.0)	4.7 (0.9)	3.3 (0.8)	2.7 (0.8)
Ishii et al. [14]	3.7 (2.0)	3.5 (1.4)	3.3 (1.0)	4.3 (1.4)	5.7 (1.9)
Our study	–	3.9 (2.0)	4.2 (2.9)	4.1 (3.0)	4.1 (2.8)

### COR for maximum flexion / extension

**Fig. 3:**
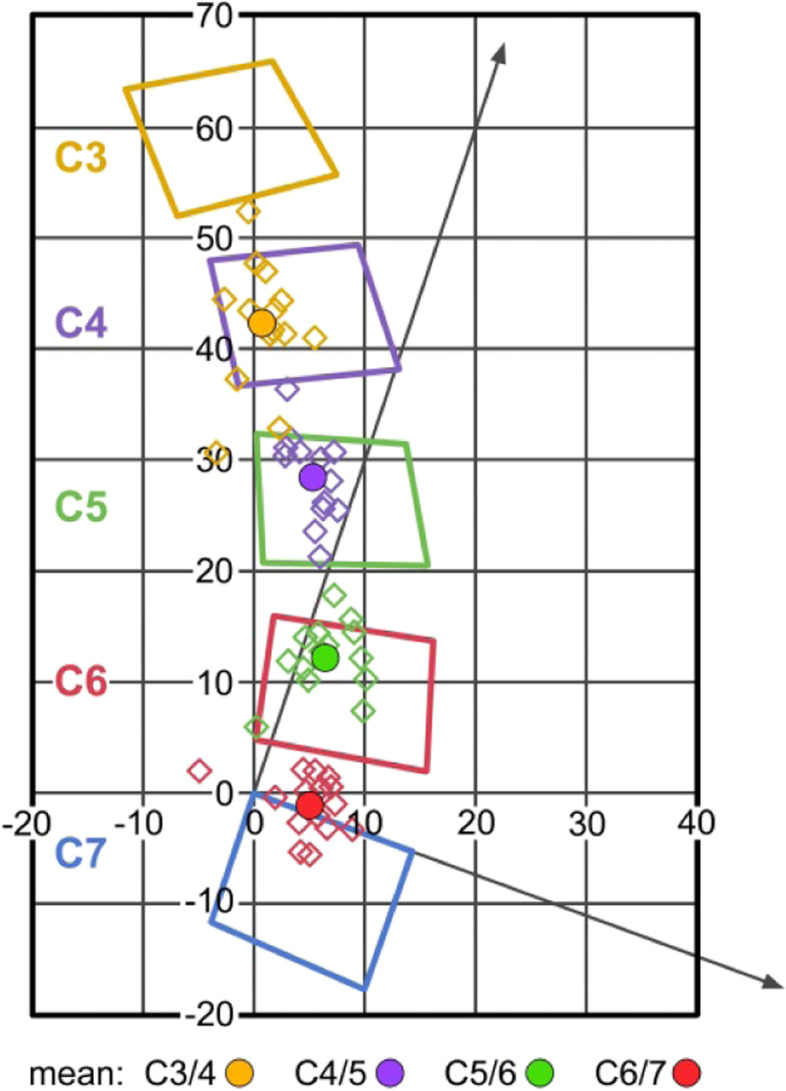
COR for maximum flexion / extension (schematic illustration)

The following coordinates (mean, SD) were found for flexion / extension: C3/4: x4.8 / y-5.8 (2.3 / 5.6); C4/5: x4.8 / y-3.8 (2.3 / 3.7); C5/6: x4.8 / y-4.0 (2.8 / 3.1); C6/7: x4.9 / y-1.1 (3.2 / 2.6). Figure 3 graphically illustrates the data found for the CORs in flexion / extension (schematic illustration) and demonstrates that the COR is located within the lower vertebral body of the motion segment, but it gradually changes its coordinates towards a position closer to the upper endplate from C3/4 to C6/7.

### Change of the COR during flexion / extension

In all 15 volunteers, the respective CORs for the 4 separate flexion/extension-intervals were calculated and compared with the coordinates for the respective CORs for maximum flexion / extension. The CORs for ME-IE, IE-N, N-IF and IF-MF are summarized in Table 3.

**Table 3:** CORs flexion/extension for the respective intervals ME-IE, IE-N, N-IF and IF-MF compared to the COR for maximum flexion/extension

Level	COR	x					y				
		ME-IE	IE-N	N-IF	IF-MF	ME-MF	ME-IE	IE-N	N-IF	IF-MF	ME-MF
C 3/4	mean	8.1	1.4	−4.2	5.0	4.8	0.8	−4.2	−10.4	−1.9	−5.8
	SD	9.1	3.1	13.6	7.4	2.3	13.4	2.5	9.4	7.9	5.6
	P	0.251	**0.005**	0.066	0.949		0.131	0.337	0.187	0.755	
C 4/5	mean	5.3	1.8	−0.2	6.0	4.8	1.0	−4.4	−7.0	−5.3	−3.8
	SD	12.5	4.7	8.2	11.5	2.3	11.0	9.0	5.0	15.1	3.7
	P	0.901	**0.049**	**0.040**	0.728		0.212	0.842	0.067	0.788	
C 5/6	mean	9.4	4.7	1.1	10.7	4.8	−3.4	−3.2	−7.5	−1.3	−4.0
	SD	5.9	4.7	5.4	13.6	2.8	8.2	2.8	4.9	6.4	3.1
	P	**0.026**	0.948	**0.035**	0.163		0.792	0.486	**0.034**	0.243	
C 6/7	mean	5.5	−0.3	6.8	8.9	4.9	−6.0	−6.9	0.3	−8.0	−1.1
	SD	7.5	9.2	3.3	15.3	3.2	11.6	8.4	8.8	6.9	2.6
	p	0.845	0.074	0.125	0.430		0.314	**0.033**	0.573	0.404	

*ME* maximum extension, *IE* intermediate extension, *N* neutral, *IF* intermediate flexion, *MF* maximum flexion

In all investigated levels the respective COR did not remain constant during flexion / extension but changed its coordinates.

**Fig. 4:**
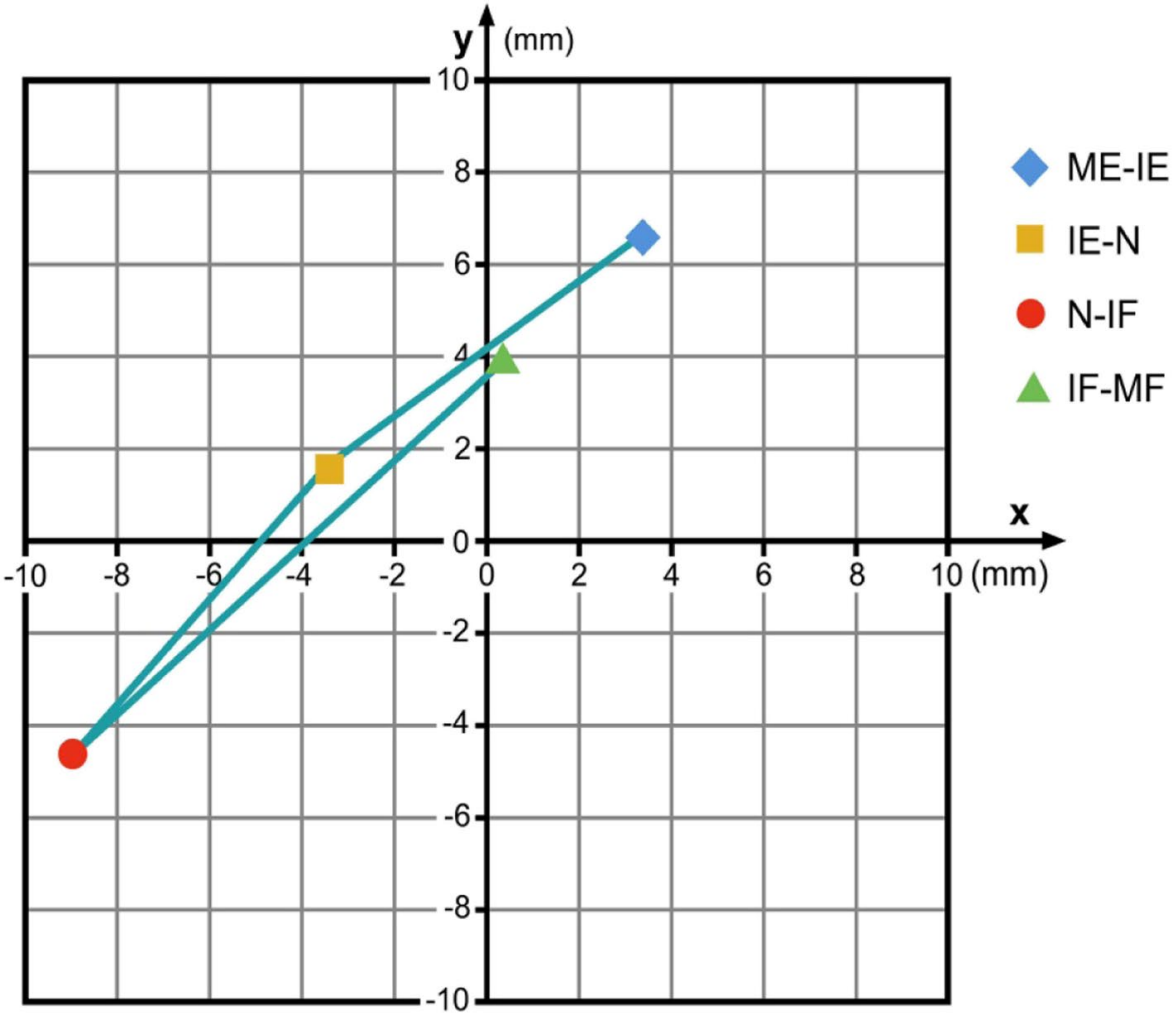
COR flexion/extension C3/4 for the respective intervals ME-IE, IE-N, N-IF and IF-MF in relation to the COR for maximum flexion / extension. Coordinate-origin 0/0 represents the COR for maximum flexion/extension. (ME: maximum extension; IE: intermediate extension; N: neutral; IF: intermediate flexion; MF: maximum flexion)

Level C3/4: from ME-IE there is no significant difference for the x- and the y-coordinates (x: p = 0.251; y: p = 0.131); from IE-N a significant difference was found for the x-coordinates (x: p = 0.005) but not for the y-coordinates (y: p = 0.337); from N-IF a near-significant difference for the x-coordinates was found (x: p = 0.066) but not for the y-coordinates (y: p = 0.187); from IF-MF no significant difference was found both for the x- and y-coordinates (x: p = 0.949; y: p = 0.755). Figure 4 illustrates the data found for C3/4.

**Fig. 5:**
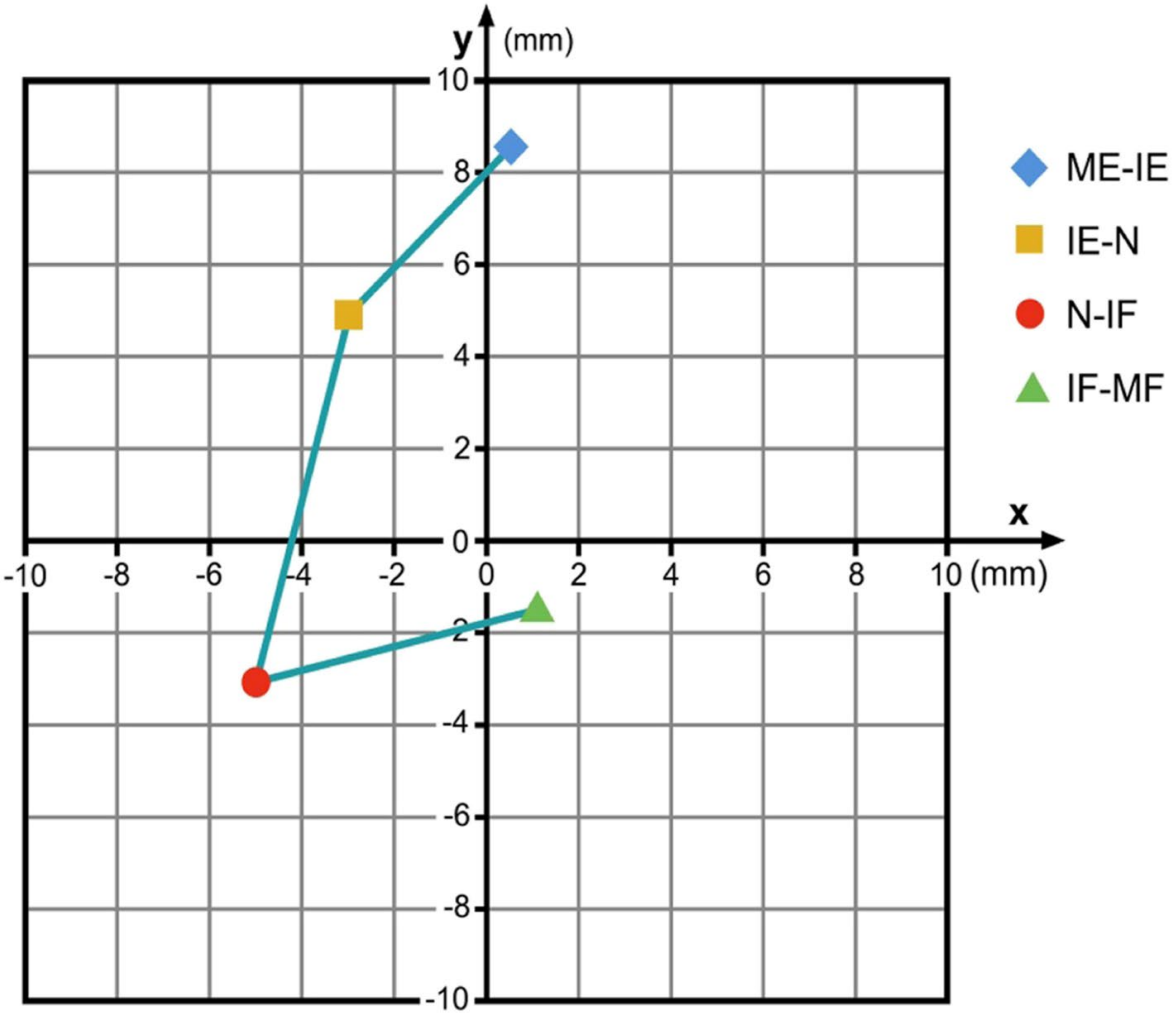
COR flexion/extension C4/5 for the respective intervals ME-IE, IE-N, N-IF and IF-MF in relation to the COR for maximum flexion / extension. Coordinate-origin 0/0 represents the COR for maximum flexion/extension. (ME: maximum extension; IE: intermediate extension; N: neutral; IF: intermediate flexion; MF: maximum flexion)

Level C4/5: from ME-IE, no significant difference both for the x- and the y-coordinates was detected (x: p = 0.901; y: p = 0.212); from IE-N a significant difference was found for the x-coordinates (x: p = 0.049) but not for the y-coordinates (y: p = 0.842); for N-IF a significant difference for x- and a near-significant difference for the y-coordinates was detected (x: p = 0.040; y: p = 0.067); the final interval from IF-MF reveals a non-significant change both for the x- and the y-coordinates (x: p = 0.728; y: p = 0.788). Figure 5 illustrates the data found for C4/5.

**Fig. 6:**
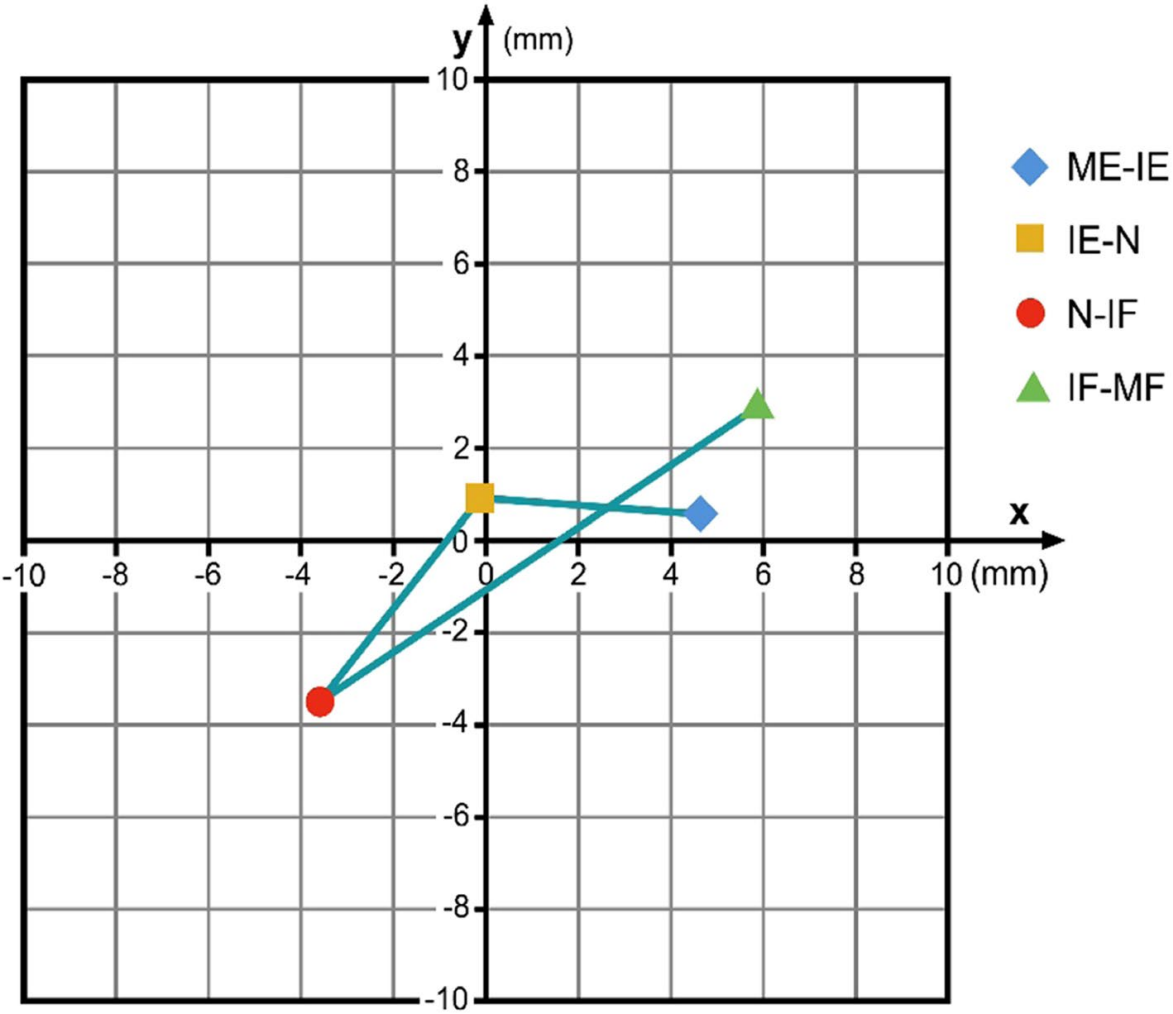
COR flexion/extension C5/6 for the respective intervals ME-IE, IE-N, N-IF and IF-MF in relation to the COR for maximum flexion / extension. Coordinate-origin 0/0 represents the COR for maximum flexion/extension. (ME: maximum extension; IE: intermediate extension; N: neutral; IF: intermediate flexion; MF: maximum flexion)

Level C5/6: From ME-IE the x-coordinates differ significantly (x: p = 0.026) but not the y-coordinates (y: p = 0.792); from IE-N no significant difference was found both for the x- and y-coordinates (x: p = 0.948; y: p = 0.486); from N-IF, significant differences were detected both for the x- and for the y-coordinates (x: p = 0.035; y: p = 0.034); for the final interval from IF-MF no significant differences could be found (x: p = 0.163; y: p = 0.243). Figure 6 illustrates the data found for C5/6.

**Fig. 7:**
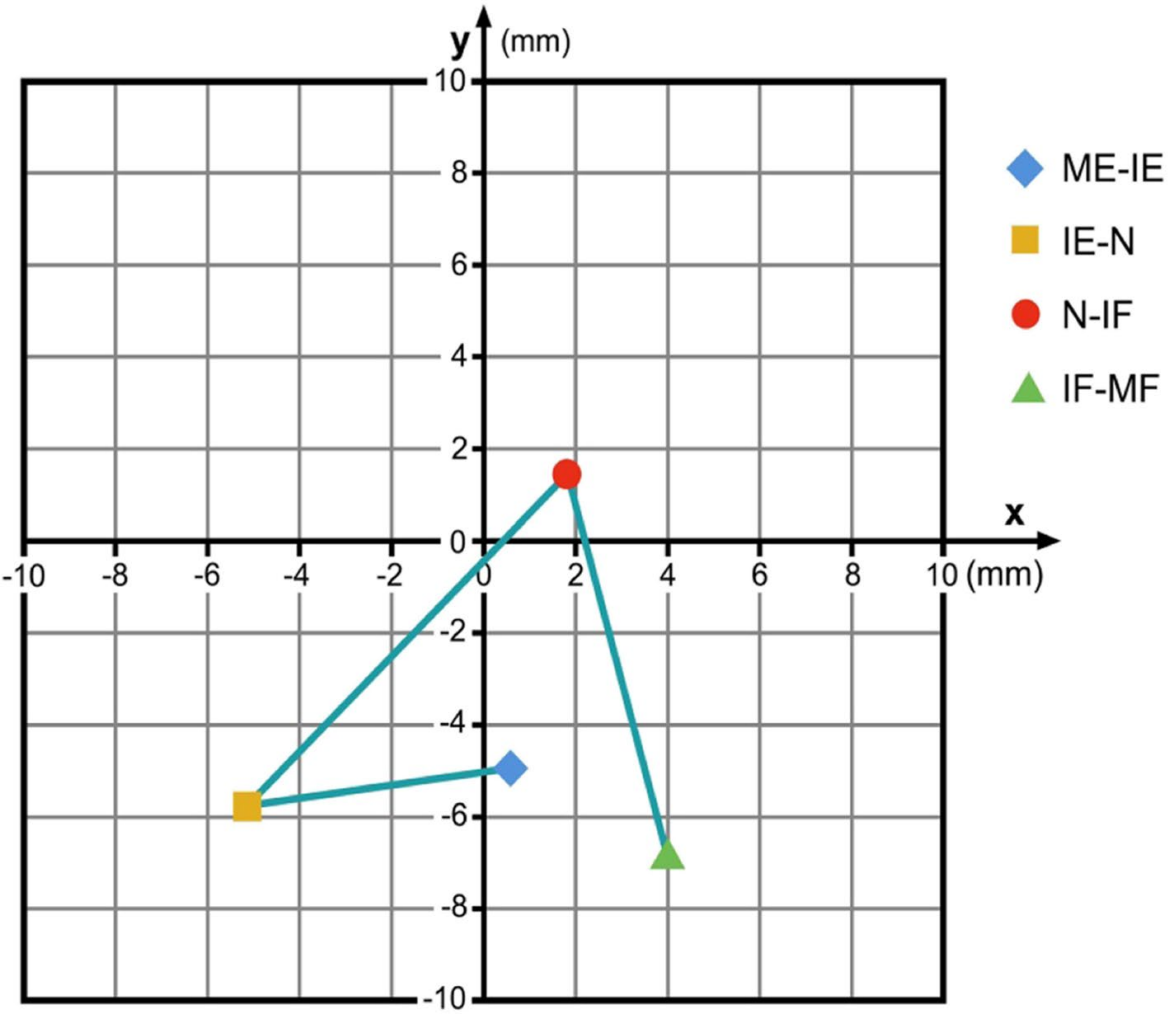
COR flexion/extension C6/7 for the respective intervals ME-IE, IE-N, N-IF and IF-MF in relation to the COR for maximum flexion / extension. Coordinate-origin 0/0 represents the COR for maximum flexion/extension. (ME: maximum extension; IE: intermediate extension; N: neutral; IF: intermediate flexion; MF: maximum flexion)

Level C6/7: No significant differences for the x- or y-coordinates were found for the ME-IE-interval (x: p = 0.845; y: p = 0.314); from IE-N the x-coordinates did not change significantly (x: p = 0.074), however a significant difference for the y-coordinates was detected (y: p = 0.033); for the N-IF-interval a non-significant difference was found both for the x- and y-coordinates (x: p = 0.125; y: p = 0.573); for the final interval IF-MF no significant changes for x- or y-coordinates were detected (x: p = 0.430; y: p = 0.404). Figure 7 illustrates the data found for C6/7.

This data demonstrates that flexion / extension in the lower cervical spine cannot be described as a simple orbit defined by a constant COR. From C3/4 to C5/6, a similar motion-path for the COR during flexion / extension can be identified: During the ME-IE, the IE-N and also the N-IF intervals the COR migrates caudally and posteriorly. During the IF-MF interval the COR moves again cranially and anteriorly. The change of the y-coordinates for the respective CORs demonstrates that from C3/4 to C5/6 the upper vertebral body of the respective motion segment rotates with a smaller radius at the final motion parts during a flexion / extension maneuver than during the intermediate part of this motion. The reasons why the coordinates for the C6/7-COR migrate in a different manner than the CORs from C3/4 to C5/6 however cannot be clearly answered with this data and requires further investigation.

### COR for lateral bending

**Fig. 8:**
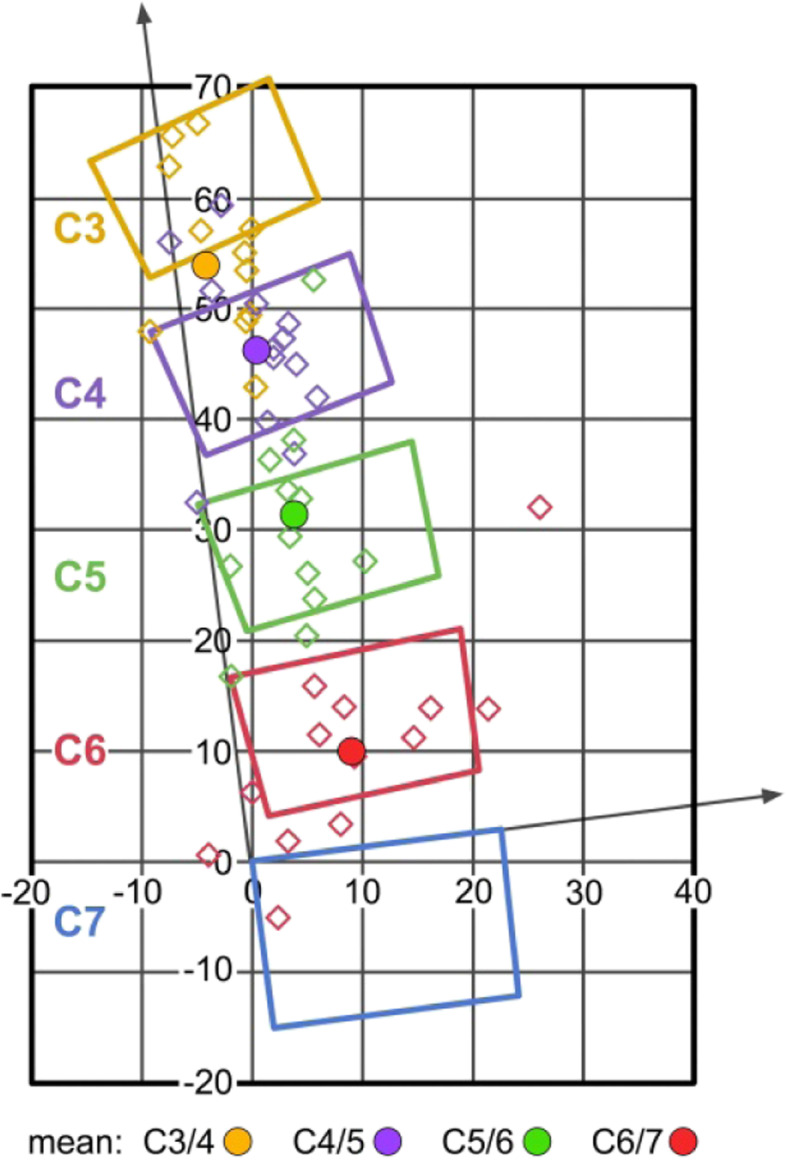
COR for maximum lateral bending (schematic illustration)

The following coordinates (mean, SD) were found for maximum lateral bending: C3/4: z-7.0 / y8.1 (3.2 / 7.3); C4/5: z-5.9 / y14.9 (3.8 / 6.4); C5/6: z-6.1 / y15.9 (2.9 / 9.3); C6/7: z-9.1 / y9.8 (8.6 / 9.2). Other than in flexion / extension, the COR for lateral bending is located within the upper vertebral body of the respective motion segment (Fig. 8).

### Change of the COR during lateral bending

From neutral to maximum left-bending, the following coordinates for the respective CORs were found: C3/4: z − 6.8 / y7.3 (5.1 / 6.8); C4/5: z-8.0 / y10.5 (5.3 / 6.8); C5/6: z-5.8 / y13.4 (7.2 / 7.9); C6/7: z-4.4 / y9.4 (13.6 / 9.9). Table 4 summarizes the respective coordinates; compared with the COR for maximum lateral bending. At no level significant differences for the z- and y-coordinates could be detected; moreover, SD was high in all data-sets.

**Table 4:** CORs lateral bending for ML-N compared to the COR for maximum lateral bending ML-MR (ML: maximum left; MR: maximum right; N: neutral)

Level	COR	z		y	
		ML-N	ML-MR	ML-N	ML-MR
C 3/4	mean	-6.8	−7.0	7.3	8.1
	SD	5.1	3.2	6.8	7.3
	P	0.928		0.780	
C 4/5	mean	−8.0	−5.9	10.5	14.9
	SD	5.3	3.8	6.8	6.4
	P	0.286		0.120	
C 5/6	mean	−5.8	−6.1	13.4	15.9
	SD	7.2	2.9	7.9	9.3
	P	0.890		0.483	
C 6/7	mean	−4.4	−9.1	9.4	9.8
	SD	13.6	8.6	9.9	9.2
	p	0.303		0.925	

### Implications for the design of cervical disc prostheses

Our data shows that in order to resemble natural motion, cervical arthroplasty devices need 2 separate and independent CORs for flexion / extension and lateral-bending. The COR for flexion / extension should be located below disc level and should be able to change its position during motion both along the x- and the y-axes, so it fits the different locations of the CORs at the respective segments, it allows smaller and larger radii, and it allows radius change during motion to facilitate tilting at the final parts of flexion / extension. Traveling of the COR along the x-axis allows translation in combination with rotation.

The separate COR for lateral-bending should be located above disc level, it need not be variable, but it must allow rotation through an oblique sagittal axis (ascending from anterior to posterior) in order to facilitate coupled motion of lateral-bending with rotation.

**Fig. 9:**
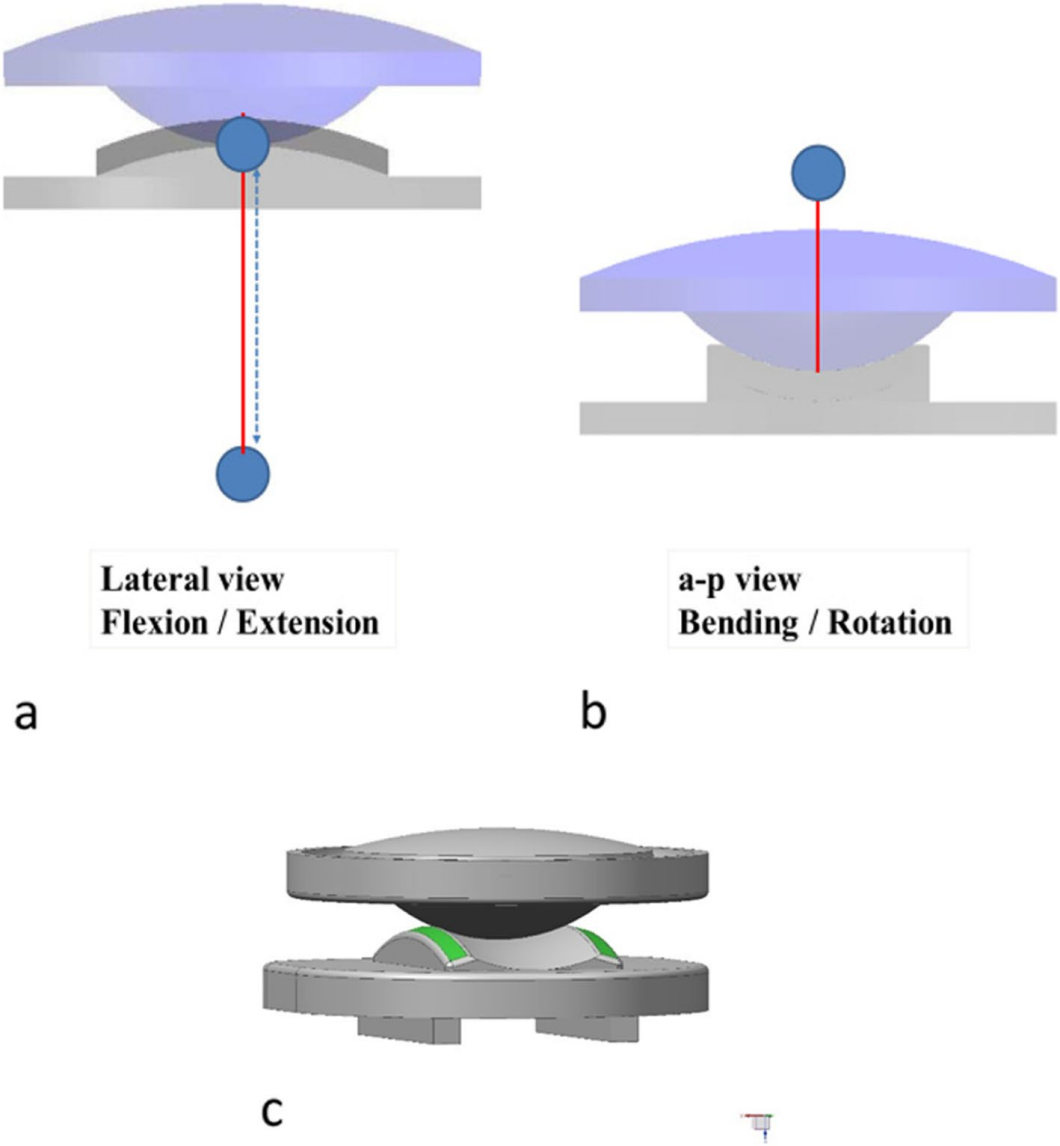
Biomechanical concept and possible design of a simple 2-piece cervical disc prosthesis fulfilling the biomechanical requirements described in our study **a** Flexion / extension is facilitated through a variable radius also allowing sagittal translation and tilting when necessary **b** Separate COR for lateral-bending above disc level allowing coupled motion with physiological ipsilateral rotation **c** Total view showing the saddle-like gliding surface of the lower part and the spherical gliding surface of the upper part of the prosthesis

Figure 9 schematically depicts such a biomechanical concept sketching a device which is absolutely simple and only needs 2 gliding partners – e.g., the upper and lower part of a disc prosthesis – and fulfills the previously described requirements. The lower gliding surface is shaped like a saddle with a convex surface in the ap-direction and a greater radius, and a concave surface in the lateral direction and a smaller radius. The upper gliding part has a convex spherical surface with the same radius fitting into the concave rim of the saddle. While traveling along the saddle in ap-direction plus rotating inside the saddle, the COR for flexion / extension is completely variable; lateral-bending is facilitated by lateral rotation inside the saddle through the separate COR above the disc. This also allows coupled ipsilateral rotation and guidance through the unco-foraminal joints.

## Discussion

This study showed that the COR for flexion / extension is located below disc level, but not at a stationary position and changes its position over the motion period. Flexion / extension is not a circular motion but is facilitated through a completely variable COR. For lateral bending a separate COR was found above disc level. This COR remains constant throughout motion and is located at an oblique sagittal axis, therefore allowing coupled lateral bending and rotation.

Cervical disc prostheses are used to preserve motion after discectomy. It is generally accepted that they should preferably resemble physiological motion as close as possible. Nevertheless, they come in a great variety of completely different biomechanical concepts. At least at the beginning, the basic idea was to use ball-socket designs understanding that flexion / extension is a circular motion guided by the curvature of the facet joints. But even if the surface of the facet joints roughly appears spherical and therefore might determine a circular motion [28], these joints can only guide motion to a certain extent, but they cannot function as a rigid rail and force the vertebral bodies in a strictly circular track. Moreover, it is not a simple torque which is applied to the cervical vertebral bodies that causes flexion / extension, but rather a complex symphony of forces including sagittal translation, axial compression and/or tension together with rotation. And there is a considerable inter-individual variety both for the anatomy but especially for the strength of the muscles and the ligaments which finally initiate and guide cervical spine motion. Therefore, the concept of simple circular motion is not suitable anymore in cervical arthroplasty, and research on qualitative motion must be translated into better biomechanical design of these devices.

A considerable number of studies investigated the ROM of the cervical spine (Tables 1 and 2) [2, 4, 6, 15, 17, 25, 27]. The ROM for flexion / extension found in our study is within the lower range of previously published data [2, 4, 17, 25, 27], probably due to the MRI investigation technique, where the volunteers must remain in maximum flexion and extension for a longer period of time than for functional X-rays. The ROM for lateral bending found in our study is slightly below the values reported in the literature for studies referring to functional X-rays [6, 25, 29], but it is similar to the values that were reported from a functional-MRI-study [15] .

Less information is found in the literature regarding the COR. Studies published by Penning, Amevo and van Mameren [9, 16, 18, 19] contribute to the understanding of the motion pattern of the cervical spine. Especially the scientific work from Bogduk [17] reveals that the COR for flexion / extension is found at different locations from C3/4 down to C6/7, and that there is a separate COR for lateral-bending located more superiorly than the COR for flexion / extension. The data found in our study is congruent to these findings, but more than that it describes that – and how - the COR for flexion / extension changes its position during motion.

Frobin described flexion / extension as a combined rotation and translation [30], but still regarded it as a circular motion following an orbit with a given COR.

Van Mameren presented a biomechanical analysis based on data derived from a cineradiographic study: 25 radiographic frames were taken during flexion / extension and separately analyzed [9]. However, also this study does not reveal whether the COR remains constant during motion.

Anderst and Baillargeon contributed in their studies to a better understanding on 3D-motion of the cervical spine during flexion / extension, lateral bending and rotation [20–22]; Flexion / extension is described as motion following the sagittal plane with the COR (centrode, as it is called in his studies) being both level-dependent and showing translation during motion, but the path of the COR is finally not illustrated in a manner that could easily be translated into an improved disc-prosthesis design. In our work we describe the respective positions of the CORs together with their motion paths during flexion / extension, therefore we believe that our work is more illustrative for a discussion how a disc prosthesis could be designed in order to replicate this COR motion path during flexion extension.

For lateral bending and rotation Anderst precisely described coupled motion in his 3D-analysis. However, this study shows that lateral bending and rotation are not two separate complex 3D-motions, but they rather appear as one relatively constant rotation around a sagittal oblique axis. The steeper the angle of this axis, the higher is the rotational component, a flat angle - as it is found in the lower cervical motion segments – causes a higher bending component than rotational component [22]. Therefore, when investigating whether the COR changes its position during lateral bending, also 2D-analysis can reliably answer this question: the intersection-point between this oblique sagittal axis with a frontal plane (the plane where the respective frontal MRI pictures were taken) remains independent from coupled rotation. Therefore it can be detected in 2D-analysis whether the COR for lateral bending remains constant or changes its position during motion. However, it should be mentioned that an oblique rotational axis does not allow anymore to give y-coordinates for a COR for lateral bending at all, because – other than in flexion / extension where the rotational axis is strictly perpendicular to the sagittal plane – the y-coordinates are found along the oblique rotational axis and therefore are dependent from the respective x-coordinates where the frontal plane cuts this oblique axis. Therefore, the respective y-coordinates given in our data are reliable for detecting changes of the COR for lateral bending, but they do not define its actual position.

Our study demonstrates that flexion / extension is not a simple circular motion following an orbit defined by one single constant COR, but the radius for the rotational component in flexion / extension varies within the respective motion segments during motion in addition to the already known segment-dependent decrease from C3/4 down to C6/7.

Figures 4, 5, 6 and 7 illustrate the migration of the COR during flexion / extension in the respective motion segments. From C3 to C6 the respective upper vertebral body mainly translates with little rotation from IE via N to IF, and then it mainly rotates with less translation from ME to IE and from IF to MF. We suppose that during the intermediate part of flexion / extension there is mainly smooth gliding following a greater radius guided by the surface of the facet joints, and that the final part of this motion is facilitated by higher muscle strength and therefore can cause tilting or other non-orbital motion influenced by the increasing tension of the joint capsules and the ligaments together with a compression of the disc. The motion pattern at C6/7 is different; the reason for this is not clear, it might be contributed to the fact that the COR for C6/7 is located closer to the upper endplate of C7 than in the motion segments above.

For the COR in lateral-bending we found no significant changes for the respective CORs during this type of motion. We suppose that the uncovertebral joints together with the facet joints function as a more rigid guidance for lateral bending and therefore keep the COR more constant than in flexion / extension.

### Limitations

The major limitation of this study is the small number of volunteers. However, many other studies about biomechanics in cervical arthroplasty published in the literature have similar small cohorts [31–36]. It is only the big IDE-studies that investigated more than 100 patients in each cohort, but these studies are sponsored by the respective companies. Our study is completely independent from any company, therefore the small number of 15 volunteers was chosen as we found that other studies also investigated patient cohorts between 15 and 20 subjects [32, 34, 36]. Although strong conclusions may not be drawn because of this limitation, we believe that our study still can contribute towards a better understanding on biomechanics with respect to cervical arthroplasty, and hopefully it will encourage colleagues to further investigate this topic with a larger cohort if possible.

We used MRI for data acquisition and manual digitizing for biomechanical calculation. We are aware that there are more precise techniques, using biplanar radiography plus high resolution CT, for instance, resulting in sub-millimeter precision [21]. However, such techniques lead to a radiation exposure of approx. 4 mSv, which is a considerable burden to healthy volunteers.

For data processing from MRI pictures to AutoCAD software, marking of the 4 corners of the vertebra was performed by a single person. Intra-rater and inter-rater variability of these markings were not determined, which can lead to variability in the COR locations. However, we expect that pooling of the data from the 15 subjects will reduce this variability.

We are also aware that our coordinates were calculated in millimeters and not as a percentage of the respective vertebral body dimension and therefore do not take into account the individual differences of the size of the volunteers‘different vertebral bodies. As shown in Table 3, the x-coordinates for maximum flexion / extension were always found in the posterior third of the respective vertebral bodies, this considerably reduces the possible error; another limitation is that no quantification of eventual degenerative changes in the asymptomatic volunteers cohort was done, which could possibly influence the coordinates of the COR. Therefore, we cannot claim to present a database defining with sub-millimeter precision where the respective CORs are exactly located in the cervical spine, but we believe that the possible resulting error from this limitation has only little influence on our findings that the COR for flexion / extension changes its position throughout motion.

The ROM in our study is in the lower range of previously published data referring to functional x-ray, but even very sophisticated other studies [21] did not include data analysis from the ends of the ROM but used the mid-range of motion. Also, the 4 intervals used for investigating the path of the COR during flexion / extension were not precisely determined but derived from the individual head-position of the respective volunteer. Therefore, these COR coordinates represent an average-interval, but we believe our data still allows a reliable description how the COR moves during flexion / extension as it was never described before in the literature.

Even if we cannot provide coordinates in sub-millimeter precision, we believe the data received from our study is still sufficiently valid to conclude how the biomechanical design of disc prostheses can be further improved.

In summary, our study showed that in flexion / extension the CORs of the investigated intermediate flexion / extension-intervals differ from the COR of the respective maximum flexion / extension for all levels from C3/4 to C6/7. Thus, the study showed that the COR is located below disc level, but not at a stationary position and changes its position over the motion period. Comparing with literature and our findings, flexion / extension is not a simple circular motion. For lateral bending a separate COR was found above disc level. This COR remains constant throughout motion and is located at an oblique sagittal axis, therefore allowing coupled lateral bending and rotation. We believe these findings can influence the design of cervical disc prostheses in future. Simple ball-socket design does not allow physiological motion; however, even simple 2-piece devices – as shown in our work – can replicate physiological motion provided the gliding partners are designed according to the biomechanical findings we presented.

## Conclusions

In order to more closely replicate in-vivo motion arthroplasty devices should have 2 independent CORs, one on either side of the disc level: For flexion / extension a variable COR below disc level that enables the upper vertebral body to rotate with flexible radii together with translation during the middle part and tilting during the end part of flexion / extension. For lateral bending the COR should be above disc level, and lateral-bending should be facilitated through an oblique sagittal axis therefore allowing coupled ipsilateral rotation, and the angle of this axis should be variable for the respective segments increasing from approx. 20 degrees for C6/7 to approx. 40 degrees for C3/4.

## Data Availability

All data are available from the corresponding author upon request.

## Abbreviations

COR: Center of Rotation
COR-HV: Center of Rotation in Healthy Volunteers
ROM: Range of Motion
MRI: Magnetic Resonance Imaging
